# Development of Crosslinker-Free Polysaccharide-Lysozyme Microspheres for Treatment Enteric Infection

**DOI:** 10.3390/polym15051077

**Published:** 2023-02-21

**Authors:** Shuo Li, Li Shi, Ting Ye, Biao Huang, Yuan Qin, Yongkang Xie, Xiaoyuan Ren, Xueqin Zhao

**Affiliations:** Zhejiang Provincial Key Laboratory of Silkworm Bioreactor and Biomedicine, College of Life Sciences and Medicine, Zhejiang Sci-Tech University, Hangzhou 310018, China

**Keywords:** lysozyme, carboxymethyl starch, chitosan, layer-by-layer self assembly, enteric infection

## Abstract

Antibiotic abuse in the conventional treatment of microbial infections, such as inflammatory bowel disease, induces cumulative toxicity and antimicrobial resistance which requires the development of new antibiotics or novel strategies for infection control. Crosslinker-free polysaccharide-lysozyme microspheres were constructed via an electrostatic layer-by-layer self-assembly technique by adjusting the assembly behaviors of carboxymethyl starch (CMS) on lysozyme and subsequently outer cationic chitosan (CS) deposition. The relative enzymatic activity and in vitro release profile of lysozyme under simulated gastric and intestinal fluids were investigated. The highest loading efficiency of the optimized CS/CMS-lysozyme micro-gels reached 84.9% by tailoring CMS/CS content. The mild particle preparation procedure retained relative activity of 107.4% compared with free lysozyme, and successfully enhanced the antibacterial activity against *E. coli* due to the superposition effect of CS and lysozyme. Additionally, the particle system showed no toxicity to human cells. In vitro digestibility testified that almost 70% was recorded in the simulated intestinal fluid within 6 h. Results demonstrated that the cross-linker-free CS/CMS-lysozyme microspheres could be a promising antibacterial additive for enteric infection treatment due to its highest effective dose (573.08 μg/mL) and fast release at the intestinal tract.

## 1. Introduction

Microbial infections, such as inflammatory bowel disease (IBD), threaten the health of human beings globally, and are associated with chronic inflammation, cancer, high mortality and so on [1]. The spread of antibiotic-resistant bacteria requires the development of new antibiotics or novel strategies for infection control [2,3]. Bioactive proteins extracted from natural products have caught more and more attention, due to their safety and few side effects compared with synthetic drugs [4,5,6,7]. Lysozyme, easily obtained from egg whites, is a glycoside hydrolase with high enzymatic specificity for the hydrolysis of β 1-4 glycosidic bonds in chitin or the peptidoglycan wall of fungal or Gram-positive bacteria, which can eventually induce cell lysis without involving antibiotic resistance [7]. Lysozyme is important for the resolution of inflammation at mucosal sites in the human gastrointestinal (GI) tract [8]. Thus, lysozyme has become a new antibacterial alternative for the treatment of IBD because of its innate activity [9], and its usage is limited by the harsh environment of the GI tract and its inherent limitations such as poor stability, low bioavailability and a narrow antimicrobial spectrum [10]. The development of immobilized lysozyme is an urgent need in medicine, the food industry and biotechnology.

The microencapsulation technique allows the isolation of active protein into solid matrices for the stabilization of active ingredients, enhancement of the bioavailability, responsive release, etc. [11]. Biopolymers, especially polysaccharides, are well-developed as the matrix to fabricate microspheres due to their excellent biocompatibility, biodegradability and low cost [12,13,14,15,16,17].Carboxymethyl starch (CMS) is a negatively charged ether derivative of starch with good water solubility and has become a suitable substrate for enzymatic immobilization. The presence of polar carboxylic functional groups in CMS grants the microspheres with mucoadhesive properties, pH sensitivity and digestion resistibility depending on the cross-linking and the degree of substitution [18,19,20,21]. However, the CMS-entrapped protein may be denatured by the proteolytic enzymes due to the rapid dissolution of CMS in an intestinal tract environment. Combining CMS with a second polymer further improves stability. In fact, chitosan (CS) as another polysaccharide can form complexes with CMS via electrostatic interaction for protein immobilization, particularly for oral administration [21,22,23,24]. For instance, CS/CMS-coated microspheres that were fabricated largely retained lysozyme under simulated stomach conditions and achieved the release of lysozyme under simulated intestinal conditions [23]. The CS/CMS polyelectrolyte complex enhanced the entrapment of bovine serum albumin (BSA) and release time (72 h) compared with CS-tripolyphosphate [24]. However, in most reports available, the protein-loaded CMS/CS preparation requires permanent chemical cross-linking or surfactants to obtain stable nanoparticles. On the other hand, the rapid release of antibacterial agents at the site is important for infection treatments. Considering the muco-adhesiveness and lysozyme triggered degradability of CS, we envisage that controlling the assembly behaviors of CMS on lysozyme, and in turn adjusting the outer CS deposition, can tailor the stability and release properties of lysozyme to enhance antimicrobial ability in enteric infections.

Herein, CMS and CS were employed as edible materials for lysozyme entrapment to treat enteric infection via layer-by-layer self-assembly without any surfactant and cross-linker. Different surface potential-contained CMS-lysozyme particles were first obtained and then deposited with different CS content to prepare CS/CMS-lysozyme microspheres. The relative enzymatic activity and in vitro release profile of lysozyme under simulated gastric and intestinal fluid were investigated. Finally, the biocompatibility and in vitro antibacterial activity of microspheres were also examined.

## 2. Materials and Methods

### 2.1. Materials

Lysozyme (from eggs, 70,000 U/mg), pancreatin, chitosan (CS, viscosity < 200 mPa.s) and carboxymethyl starch sodium (CMS, MW 50KD) with a substitution degree of 0.1046 were purchased from Aladdin Co., Ltd. (Shanghai, China). *Escherichia coli* O157:H7 (*E. coli*, ATCC 25922) and *Staphylococcus aureus* (*S. aureus*, ATCC25923) were bought from Sigma–Aldrich Corp., St. Louis, MO, USA. Human umbilical vein endothelial cells (hUVECs) were purchased from the China Center for Type Culture Collection. Lysozyme assay kit and bicinchoninic acid (BCA) protein assay kit were purchased from Jiancheng Bioengineering Institute (Nanjing, China). The Luria–Bertani broth (LB Broth), RPMI1640 culture medium, fetal bovine serum (FBS), Penicillin-Streptomycin solution (10 mg/mL) and Cell Counting Kit-8 (CCK-8) were purchased from Beijing LABLEAD Co., Ltd. (Beijing, China). Ultrapure water from a Milli-Q filtration system (Millipore Corp., Bedford, MA, USA) was used to prepare all solutions.

### 2.2. Preparation of Encapsulated Lysozyme Microspheres

CS/CMS-lysozyme was prepared by layer-by-layer (LbL) assembly via ionic interaction of polysaccharides. Zeta-potential measurements and encapsulation efficiency were used to assess the optimum assembly condition. Typically, 0.1 g of lysozyme was firstly dissolved in 100 mL PBS (0.01 M, pH 3.0) to obtain a positively charged polyelectrolyte solution, then 0.3 g of CMS was mixed with the lysozyme solution gently for 2 h for the deposition of CMS on the lysozyme surface via electrostatic interaction. After centrifugation at 10,000 rpm for 10 min, the precipitate was washed with distilled water three times. Subsequently, 0.2 g of the above-obtained CMS-lysozyme microspheres was suspended in 1.6 mL of positively charged CS solution (1 mg/mL) and incubated at 25 °C for 1.5 h under stirring of 300 rpm. The CS solution was prepared in advance by dissolving 0.1 g chitosan in 100 mL 2% acetic acid solution (in PBS with 0.01 M, pH 4.0) and then pH was adjusted with hydrochloric acid (6 M). The resulting CS/CMS-lysozyme microspheres were centrifuged at 4000 rpm for 3 min and then washed with distilled water. Excessive lysozymes from all the washing solutions were collected for further determination of the lysozyme loading capacity. 

The non-encapsulated lysozyme content was quantified using the micro-BCA protein assay kit by reading the absorbance at 562 nm. All experiments were performed in triplicate, and the drug loading capacity (LC) and drug encapsulation efficiency (EE) of the obtained microspheres were calculated according to the following formula:

(1) $$\mathrm{LC}\left(\%,w/w\right)=\frac{W_{total\;lysozyme}-W_{free\;lysozyme}}{W_{totalPs}}\times 100\%$$

(2) $$\mathrm{EE}\left(\%,w/w\right)=\frac{W_{totallysozyme}-W_{freelysozyme}}{W_{total\;lysozyme}}\times 100\%$$

where $W_{total\;lysozyme}\;$ is the initial amount of lysozyme; $W_{free\;lysozyme}$ is the amount of unloaded lysozyme measured in the supernatant and washing solution; and $\;W_{totalPs}$ is the mass of the initial microsphere powders added in the system. 

### 2.3. Microsphere Characterization

The hydrodynamic sizes and zeta potentials were measured on a Nano-ZS Zetasizer dynamic light scattering (DLS) instrument (Malvern Instruments Ltd., Malvern, UK). The microstructure of the sample was measured using a field emission scanning electron microscope (SEM, ZEISS-ULTRA55; Hitachi Ltd., Tokyo, Japan) and the particle diameters were calculated using Nano Measurer 1.2 software. IR analysis and UV analysis were carried out on Nicolet 5700 FTIR spectrometer (Thermo Fisher Scientific, Waltham, MA, USA) using the KBr-disk method and Nanodrop 2000 UV–vis spectrometer (Thermo Fisher Scientific, Waltham, MA, USA), respectively.

### 2.4. Enzymatic Activity of Lysozyme

Lysozyme assay was performed using *M. lysodeikticus* cells. Briefly, 0.2 mL aliquot of diluted native lysozyme in phosphate buffer (pH 6.5) or the lysozyme released from CS/CMS-lysozyme was added to 2 mL suspension of *Micrococcus lysodeikticus* cells (0.1 mg/mL) and then incubated at 37 °C for 5 min. The turbidity of cell suspension was measured every 20 s over a 2 min period on a UV–visible spectrophotometer at 530 nm. The experiments were performed in triplicate, and the enzymatic activity was estimated as relative activity (% activity, U/U) based on a decrease in turbidity relative to the native lysozyme system.

### 2.5. In Vitro Digestibility of Lysozyme Microspheres

The in vitro releasing profile of lysozyme from CS/CMS-lysozyme microspheres was examined in pepsin-free simulated gastric fluid (SGF) and simulated intestinal fluid (SIF) with pancreatin. Simulated digestive fluids were prepared according to a previous method with little modification [20]. For the preparation of simulated gastric fluid (SGF), 36.5% (*w*/*w*) hydrochloric acid (7 mL) was diluted to 1 L with deionized water at pH 1.2. Similarly, simulated intestinal fluid (SIF) was prepared by dissolving 10 g pancreatin and 6.8 g potassium di-hydrogen phosphate into 1 L of distilled water. Then, the pH of the SIF solution was adjusted to 6.8 with 0.1 M sodium hydroxide solution. Simulated colonic fluid (SCF) at pH 7.2 was prepared by dissolving potassium di-hydrogen phosphate (3103 g) and di-potassium hydrogen phosphate (4355 g) in distilled water (1 L). 

For the simulation study, microsphere-lysozyme complexes (6.25 mg) were added to 15 mL of the dissolution medium with a shaking speed of 100 rpm at 37 °C according to standard pharmacopeia methods [23]. The microspheres were firstly incubated for 2 h in SGF and then separated by centrifugation, followed by a 6 h treatment with SIF. Afterward, the medium was replaced with SCF for an additional 4 h. At periodic intervals, 1 mL of release medium was withdrawn and replaced with the same volume of fresh medium to maintain a constant volume. These experiments were performed in triplicate. The released lysozyme content was analyzed by UV–vis spectrophotometer at 278 nm. The beginning concentration was set as 100%. All experiments were performed in triplicate, and the cumulative release (%) was calculated using the equation below:

(3) $$\mathrm{Cumulative}\;\mathrm{release}\;\left(\%,\;w/w\right)=\left(15C_{i}+\overset{i-1}{\underset{1}{\sum }}C_{i-1}\times 1\right)/M_{0}$$

where $M_{0}$ is the initial mass of lysozyme in the samples and $C_{i\;}$ is the concentration of lysozyme released at each sampling time point.

### 2.6. Antibacterial Activity

Gram-negative bacteria *Escherichia coli* (*E. coli*, ATCC 29425) and Gram-positive bacteria *Staphylococcus aureus* (*S. aureus*, ATCC25923) were cultured for 12 h in a 37 °C shaker. When the OD_600_ value of the cultured bacteria reached 0.7, CMS-lysozyme or CS/CMS-lysozymemicrospheres were added to 3 mL of the bacterial suspensions with a final concentration of 15 mg/mL. As a control, free lysozyme was also added to the suspensions to the same concentration as that of CMS-lysozyme or CS/CMS-lysozyme microspheres. After incubation at 37 °C for 20 h, 100 µL of 10-fold diluted suspensions from each test strain solution were spread onto agar plates and incubated for 20 h in shaker at 37 °C. The bacterial colonies were counted and the colony number of bacteria without treatment was used as the control. All experiments were performed in quintuplicate, and the antibacterial efficiency was calculated as follows:

(4) $$\begin{array}{c} \mathrm{Antibacterial}\;\mathrm{rate}\;\left(\%,\;\mathrm{cfu}/\mathrm{cfu}\right)\\ \;\;\;\;\;=\frac{\mathrm{cell}\;\mathrm{numbers}\;\mathrm{of}\;\mathrm{control}-\;\mathrm{cell}\;\mathrm{numbers}\;\mathrm{of}\;\mathrm{samples}}{\mathrm{cell}\;\mathrm{numbers}\;\mathrm{of}\;\mathrm{control}}\times 100\% \end{array}$$

### 2.7. Cytotoxicity

In vitro cytotoxicity was measured using a standard Cell Counting Kit-8 (CCK8) assay. The hCMEC cells were seeded into a 96-well plate at 5 × 10^3^ cells/well and incubated for 24 h until the cell adhered to the wall. Then cells were treated with serum-free medium containing CS/CMS-lysozyme microspheres. After co-culturing for 24 h, cells were washed twice with PBS, and incubated with 10 μL CCK-8 solution for 1 h. The absorbance value (OD value) at 450 nm was measured by a microplate reader. All experiments were performed in octuplicate, and the relative cell viability (%) was expressed as a percentage relative to the untreated control cells.

### 2.8. Statistical Analysis

Statistical analysis was carried out using Origin 8.5 and Microsoft Excel and SPSS version 25.0. Statistical analysis was performed using two-tailed unpaired *t*-tests for comparison. The data were obtained from at least three independent experiments and expressed as means ± standard deviations (SD). Differences were statistically significant when the *p*-values were less than 0.05. 

## 3. Results and Discussion

### 3.1. Preparation and Optimization of Encapsulation Conditions

**Figure 1:**
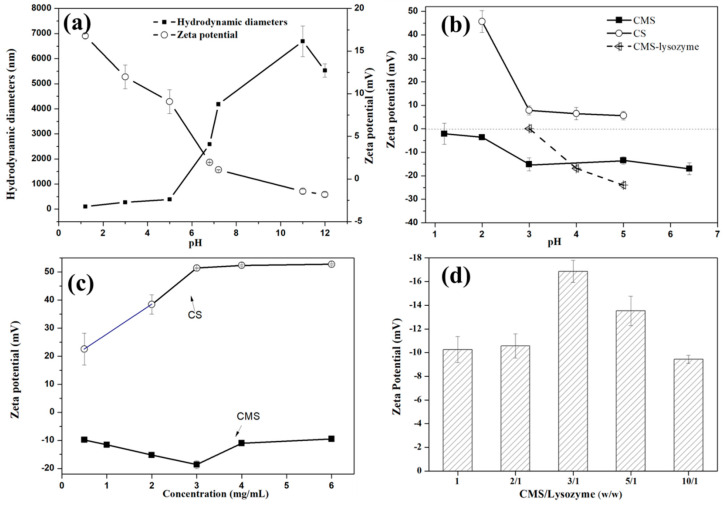
Optimization of preparation procedure according to DLS analysis: (**a**) zeta potential and size as a function of pH of lysozyme, (**b**) zeta potential as a function of pH of CMS, CS and CMS- lysozyme, (**c**) zeta potential as a function of the concentration of CMS and CS, and (**d**) zeta potential as a function of CMS-to-lysozyme mass ratio. Bars represent the corresponding standard deviations (*n* = 3).

The surface charge is one of the most important parameters tailoring the assembly behavior of polyelectrolytes during the fabrication of LBL self-assembly systems [20]. The dissociation degree of weak polyelectrolytes depends strongly on their content and the solution pH. Zeta-potential measurements were carried out to characterize the pH response behaviors of lysozyme, CS and CMS at different pH conditions. As shown in Figure 1a, CMS was nearly neutrally charged, then increased its surface negative charge with pH increase due to CMS deprotonation at the pH ranging from 1 to 6.8. CMS solution with the concentration of 3 mg/mL was found to have a maximum negative charge (−15.15 ± 2.788 mV) at pH 3, where the lysozyme molecules exhibited a positive charge (11.975 ± 1.402 mV) and smaller size (272.5 ± 6.6 nm). CMS and lysozyme were required with a relatively strong surface potential to fabricate compact structures of capsules via electrostatic interaction. When the pH was 3.0, the interaction between positively charged lysozyme molecules and negatively charged CMS molecules led to the formation of CMS-lysozyme LBL assembly capsules. As the pH value increased, the engineered CMS-lysozyme negatively charged because of CMS protonation, and exhibited a maximum opposite charge against CS at pH 4 (Figure 1b), which is the optimal pH for the assembly of the second layer. Oppositely charged polymers (CMS and CS) were electrostatically assembled directly onto the lysozyme surface to obtain CS/CMS-lysozyme microspheres. Furthermore, the substitution degrees of the CMS and the deacetylation degree of chitosan can influence their surface properties and the subsequent assembly behaviors of polyelectrolytes. To avoid the complexity of the self-assembly system, commercialized CMS and CS were selected for controlling the assembly behaviors of polyelectrolytes only by adjusting their deposition contents.

**Figure 2:**
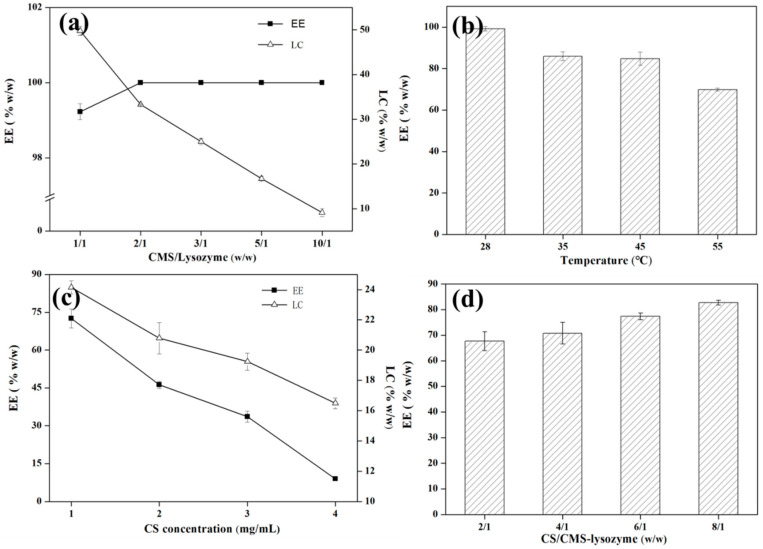
Optimization of encapsulation efficiency (EE) and loading capability (LC). EE and LC as a function of CMS-to-lysozyme mass ratio (**a**) and CS concentration (**c**). EE as a function of temperature (**b**,**d**) mass ratio of CS and CMS-lysozyme. Bars represent the corresponding standard deviations (*n* = 3).

In order to investigate the optimal encapsulation efficiency (EE), the assembly procedure was optimized by testing the key parameters such as the mass ratios of lysozyme/CMS and CMS-lysozyme/CS, CS concentration and incubation temperature. As shown in Figure 2, for the first layer, the EE was enhanced to reach a plateau with a maximum of 100% by increasing the mass ratio of CMS to lysozyme from 1:1 to 2:1, while the LC decreased significantly from 50% to 10% with the increase in the mass ratio between CMS and lysozyme from 1:1 to 10:1 (Figure 2a). Because the concentration of lysozyme in our experiment was fixed at a constant dosage of 1 mg/mL, increasing the ratio of CMS/lysozyme meant increasing the concentration of CMS. Thus, an increased CMS concentration gradient induced complete lysozyme entrapment, similar to the previous report [20]. At the same time, increased CMS/lysozyme microspheres induced LC to decrease. Considering that the highest negative charged (−16 mV, Figure 1d) CMS/lysozyme microspheres are beneficial for CS deposition, the ratio of 3/1 was selected as the optimal mass ratio of CMS/lysozyme microcapsule. For the second layer, with the increase in the CS concentration or the ratio between CS and CMS-lysozyme, both LC and EE decreased (Figure 2c,d). When the CS concentration was 1 mg/mL, EE and LC were 84.9% *w*/*w*, and 22.1% *w*/*w*, respectively. 

### 3.2. Characterization of CS/CMS-Lysozyme Microspheres

**Figure 3:**
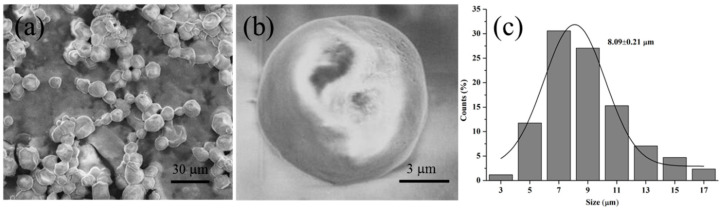
SEM morphological analysis of CS/CMS-lysozyme microspheres: (**a**) overview, (**b**) enlarged image of the individual capsule and (**c**) histograms of size distribution. The solid line in the size histograms is the simulation curve of Gaussian distribution. The data outlined refer to the most probable size.

The microstructure of the microspheres was detected by SEM. As shown in Figure 3, the microspheres exhibited a relatively spherical-shaped morphology and a relatively smooth surface with an average size of 8.09 μm.

**Figure 4:**
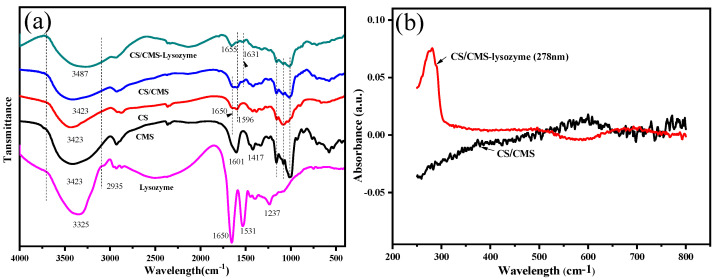
(**a**) FTIR spectra of lysozyme, CS, CMS and CS/CMS-lysozyme microspheres, (**b**) UV-visible absorption spectra of CS/CMS-lysozyme and hollow CS/CMS microspheres.

The FT-IR analysis was performed to confirm the presence and possible interactions between CS/CMS and lysozyme. As shown in Figure 4a, the broad peaks centered at 3423 cm^−1^ for CS/CMS and 3487 cm^−1^ for CS/CMS-lysozyme microspheres came from CMS (3450 cm^−1^) and CS (3443 cm^−1^), likely due to the interaction of CMS with CS via hydrogen bonding. Lysozyme incorporation induced the O–H stretching peak of CS/CMS-lysozyme to decrease and shift to the lower wavenumber, which is similar to a previous report [16]. The peaks at 1650 cm^−1^ and 1531 cm^−1^ correspond to amide I (the stretching vibration of C=O conjugated peptide bond) and amide II (the secondary NH deformation), respectively. The characteristic peaks also appeared in the CS/CMS-lysozyme (1655 cm^−1^, 1531 cm^−1^) and the CS/CMS (1654 cm^−1^, 1596 cm^−1^) FTIR spectrum, indicating the amide appearance. The CS/CMS-lysozyme spectrum showed slight discrepancies compared with the CMS/CS. The band at 1596 cm^−1^ in the CS/CMS spectrum transformed into a shoulder one due to the entrapment of lysozyme. Due to the superposition effect of the polyelectrolyte complex between the amine group of CS (1650 cm^−1^) and the carboxylate group of CMS (1601 cm^−1^) [16], the intensive peak with a higher shift to 1655 cm^−1^ was observed in the CS/CMS-lysozyme spectrum, which demonstrates intermolecular interactions and good molecular compatibility between CMS and CS. Moreover, some characteristic peaks corresponding to compound CMS, CS and lysozyme appeared in gels, further confirming the presence of lysozyme within the CS/CMS.

Monitoring wavelength changes of the UV–vis spectra can detect the formation of complexes between a protein and other macromolecules. Hence, UV–vis spectra were used to further analyze the microenvironment around microspheres. As shown in Figure 4b, CS/CMS had no absorbance from 800 cm^−1^ to 250 cm^−1^ wavelengths, while CS/CMS-lysozyme exhibited an obvious absorbance peak at 278 cm^−1^. The slight 2 nm shift from 280 nm of native lysozyme, suggests the existence and combination of lysozyme with polysaccharides in the composite microspheres [25].

### 3.3. Enzymatic Activity of the Lysozyme

**Figure 5:**
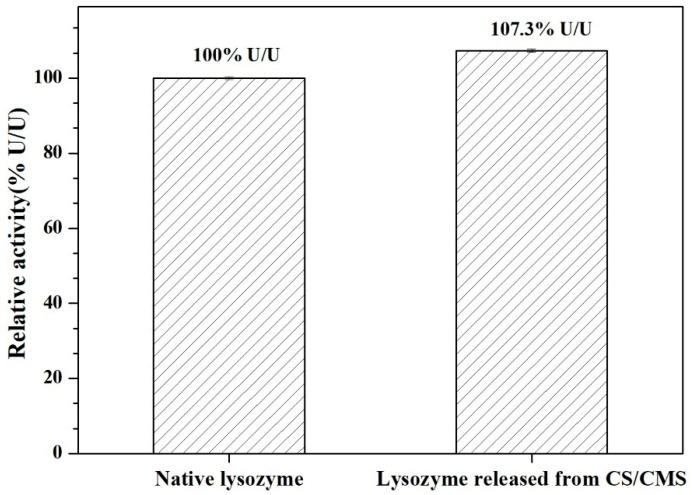
Relative activity of lysozyme released from CS/CMS. Bars represent the corresponding standard deviations (*n* = 3).

In vitro lytic activities on *M. lysodeikticus* cells of lysozymes released from microspheres are illustrated in Figure 5. The reactivity for released lysozyme was 107.4 ± 0.38% U/U, a bit higher than that of free lysozyme due to the enhancement of Na^+^ and K^+^ in the reaction solution [26,27]. Generally, lysozymes will aggregate together after release from the polymer matrix and their lytic activity may decrease due to the destabilization and unfolding of the protein [28]. It was also reported that polysaccharides and polyhydric alcohols can effectively prevent lysozymes from aggregation and inactivation [16]. Our study suggested that a mild preparation process minimized the above-mentioned defects of the polymer matrix and the CS/CMS matrix effectively protected the lysozyme.

### 3.4. In Vitro Release Studies

**Figure 6:**
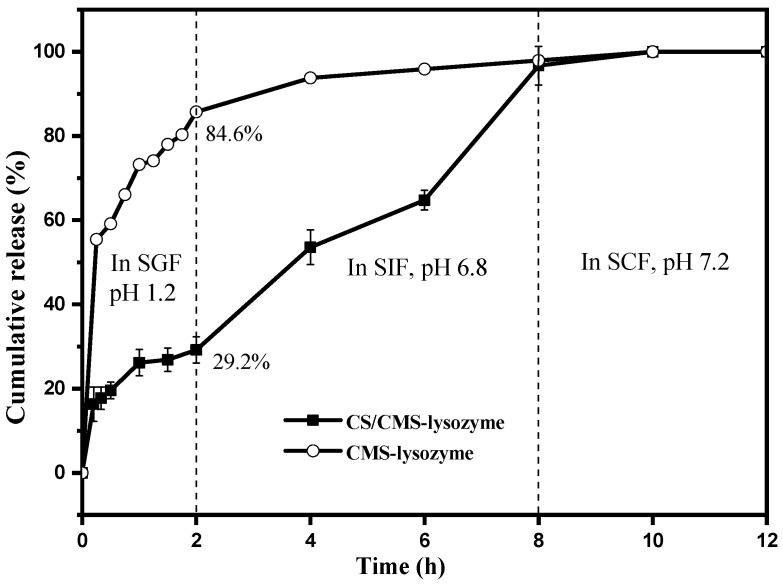
In vitro releasing kinetics of lysozyme from stabilized microspheres under stimulated digestive fluids. Bars represent the corresponding standard deviations (*n* = 3).

As the intestine is generally considered as the main absorption site, in vitro digestion and release of lysozyme from chitosan-based formulations was performed under simulated digestive fluids to investigate their intestinal targeting during the formulation. The pH and ionic strength associated with local conditions in the stomach and small intestine fluids are quite different from each other. The protective effect of polysaccharide coating is of much importance to avoid degradation of lysozyme under the condition with high acid and enzymes in SGF. As shown in Figure 6, in simulated gastric digestion the release of lysozyme from CMS-coated microspheres exhibited a burst and more than 80% of lysozyme outflowed in 2 h owing to the protonation of carboxyl groups on the CMS backbone and the sequence detachment. In addition to the pH, pancreatin and the ionic strength affected release for the CMS-based carriers depending on the degree of substitution and crosslinking. However, coating the pH-responsive CS inhibited protein release at pH 2.0 and up to 70.8% was retained in CS/CMS through the molecule–molecule aggregation at extremely acidic pH. It suggested enhanced stability in gastric fluid and the possibility of using the second polysaccharide coating of CS as a gate material. After incubation in the SGF, the microspheres were separated and transferred to SIF (pH 7.2) with pancreatin. The release of lysozyme increased significantly due to the polysaccharide degradation by pancreatin. Almost all the lysozyme was released from CS/CMS-lysozyme and the cumulative release reached 96.7% *w*/*w* in 8 h. Then, in the SCF with pH 7.2, lysozyme was completely released in 2 h and the cumulative release rate reached 100%. The absolute amount of lysozyme that eventually reached the small intestine was 573.98 μg/mL within 6 h. The results indicate that CS/CMS-lysozyme exhibited a good digestion resistance in SGF and fast release in SIF.

The pH of simulated digestive fluid modulates ionic interactions of chitosan with oppositely charged CMS and consequently the properties of microspheres. It was reported that chitosan-based formulations remained intact in the low pH gastric environment but dissociated in the small intestine with higher pH [29]. The susceptibilities of microspheres in SIF present significant effects of pancreatin. CS-coated capsules are readily degradable by pancreatin in PBS [30]. Moreover, released lysozyme can also accelerate CS degradation [31]. These results indicated that CS/CMS-lysozyme exhibited a good resistance in SGF and fast release of lysozyme in SIF.

### 3.5. Antibacterial Analysis

**Figure 7:**
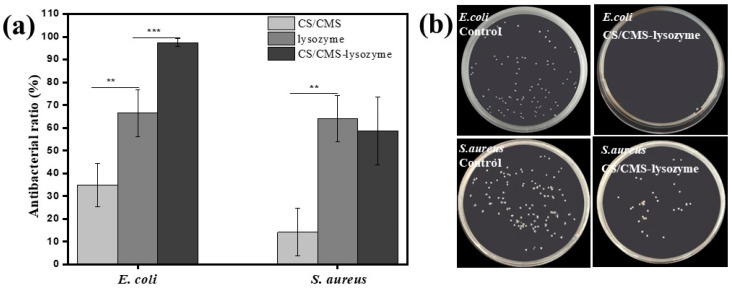
In vitro antibacterial activity: (**a**) antibacterial ratio against *E. coli* and *S. aureus*, (**b**) re-cultivated bacterial colonies on agar after bacteria dissociation from antibacterial tests. The data of antibacterial ratio were analyzed using a two-tailed unpaired Student’s *t*-test. The error bars indicate means ± SD. **: *p* < 0.01 and ***: *p* < 0.001.

Efficient antibacterial activity is essential for the antibacterial enzyme. The antimicrobial activity was quantified by counting the colony forming units (cfu) on the spread plate. As shown in Figure 7, free lysozyme has antibacterial ratios of 66.4 ± 10.4% cfu/cfu and 63.9 ± 10.2% cfu/cfu against *E. coli* and *S. aureus*, respectively. CS/CMS microspheres demonstrated a significantly enhanced antibacterial ability against *E. coli* (34.9% cfu/cfu) than *S. aureus* (14.2% cfu/cfu). Moreover, compared with the free lysozyme and hollow CS/CMS, the CS/CMS-lysozyme microspheres exhibited a better antibacterial ability against *E. coli* (97.4% cfu/cfu) than *S. aureus* (58.7% cfu/cfu) within 20 h of contact. This enhancement was attributed not only to the released lysozyme but also to a possible synergistic effect between chitooligomers and lysozyme obtained after chitosan hydrolysis [12,32,33].

### 3.6. Biocompatibility

**Figure 8:**
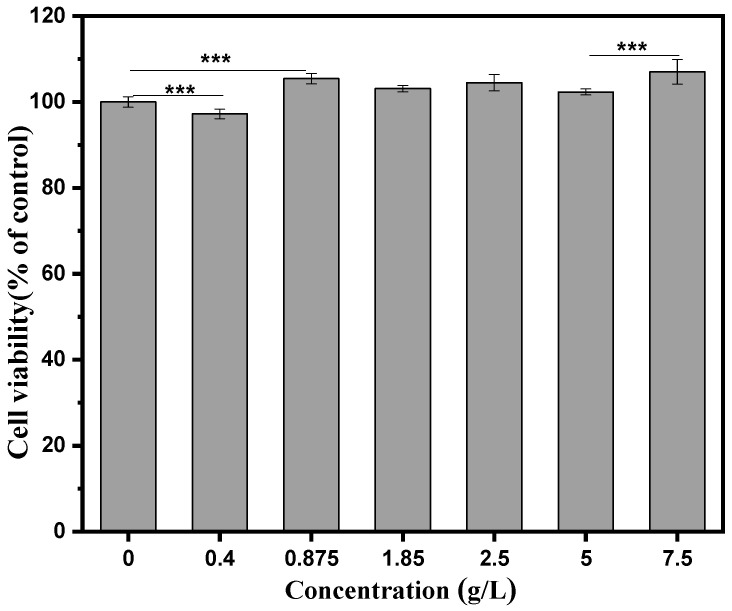
Cell viability of CS/CMS-lysozyme microspheres by CCK8 assay. The data of cell viability were analyzed using a two-tailed unpaired Student’s *t*-test. The error bars indicate means ± SD and *n* = 8. ***: *p* < 0.001.

Cytotoxicity has always been a concern for biomaterials. The cytotoxicity was determined by CCK8 assay in a concentration-dependent manner. As shown in Figure 8, the cell viability is above 100% when the tested concentrations reached 0.875 g/L, suggesting significantly improved cell proliferation.

## 4. Conclusions

Here, crosslinker-free polysaccharide-lysozyme microspheres were constructed by adjusting the assembly behaviors of carboxymethyl starch (CMS) on lysozyme and subsequently outer cationic chitosan (CS) deposition. The UV–vis and FIRT-verified entrapment of lysozyme within the CS/CMS matrix was carried out successfully. The optimized CS/CMS-lysozyme microspheres had the highest loading efficiency of 84.9% *w*/*w*, and the relative activity of the released lysozyme from CS/CMS-lysozyme microspheres was 107.4 ± 0.38% U/U compared with free lysozyme, indicating a slight promotion potency on lysozyme activity. Furthermore, 67.5% *w*/*w* of cumulative release in SIF conferred that the CS/CMS double polysaccharide layers largely retained lysozyme within the matrix without a burst in the acid environment and achieved intestine-targeted release. Moreover, the antimicrobial activity assay indicated CS/CMS-lysozyme had a higher antibacterial activity especially towards *E. coli* owing to the synergistic effect between the membrane-attacking ability of CS and the cell wall-attacking ability of lysozymes. The cross-linker-free and pH-responsive CS/CMS-lysozyme microspheres can be a promising oral antibacterial additive for the treatment of intestinal infection.

## Data Availability

The data presented in this study are available on request from the corresponding author. The data are not publicly available due to confidentiality agreements.
